# Ecotypic changes of alpine birds to climate change

**DOI:** 10.1038/s41598-019-52483-0

**Published:** 2019-11-06

**Authors:** Maria del Mar Delgado, Chiara Bettega, Jochen Martens, Martin Päckert

**Affiliations:** 1 Research Unit of Biodiversity (UMIB, UO-CSIC-PA), Oviedo University - Campus Mieres, 33600 Mieres, Spain; 2 Institut für Organismische und Molekulare Evolutionsbiologie, Johannes Gutenberg-Universität, 55099 Mainz, Germany; 3 Senckenberg Natural History Collections, Museum für Tierkunde, Koenigsbruecker Landstraße 159, 01109 Dresden, Germany

**Keywords:** Climate-change ecology, Conservation biology

## Abstract

In endotherm animals, several traits are related to climate. For example, Bergmann’s rule predicts a decrease in body size within species and across closely related species with increasing temperature, whereas Gloger’s rule states that birds and mammals should be darker in humid and warm environments compared to colder and drier areas. However, it is still not clear whether ecotypic responses to variation in the local environment can also apply to morphological and colouration changes through time in response to climate change. We present a 100-year-long time series on morphological and melanin-based colours of snowfinch (325 *Montifringilla*, 92 *Pyrgilauda* and 30 *Onychostruthus*) museum specimens. Here we show that the tarsus length of the species has decreased and the saturation of the melanin-based colour has increased, which was correlated with the increase of temperature and precipitations. As ecotypic variations are tightly linked to individual behavioural and physiological responses to environmental variations, differently sized and coloured individuals are expected to be differently penalized by global changes. This study opens the pertinent question about whether ecotypic responses can enhance population persistence in the context of global change.

## Introduction

In endotherm animal species, several body traits are related to climate. For example, following Bergmann’s rule predictions, individuals within the geographical range of a species should tend to be larger in body size under colder climate conditions^1^. Body size affects energy and water requirements for thermoregulation, such that one underlying rationale of such climate-related variation in body size is that heat conservation associated with larger body size provides benefit in cooler climates. The deposition of melanin pigmentation is also correlated with climatic variables, and Glogers’ rule states that, along climatic gradients, birds and mammals tend to be darker in humid and warm environments^2^. Darker colorations have been shown to confer benefits in warmer and humid environments for camouflage, photoprotection, protection against parasites and some other advantages linked to immunity, stress responses, basal metabolic rate and behavioural traits (i.e. some pleiotropic effects of genes involved in melanin-based colouration)^2,3^. These adaptations have resulted in the well-established ecotypic rules^4^.

Some studies have speculated on the possibility that the ecotypic responses to variation in the local environment can also apply to morphological and colouration changes through time in response to climate change^3,5,6^. Yet, ecotypic responses to climate changes are not easily predictable^7^, and evidence for morphological and colouration changes in response to climate warming is mixed^5,8,9^. For example, while some studies have stressed the possibility that body size is declining as a response to climate warming^7^, others did not fully support this hypothesis^10^. Furthermore, some evidence have suggested that the frequency of melanin-based colour morphs in many animals may be altered^6^ as a direct consequence of climate warming or indirect selection exerted on phenotypic traits that are genetically correlated with colouration. However, while Karell *et al*.^9^ showed that climate warming lead to an increase of the brown melanin-based morph in tawny owls (*Strix aluco*) due to changing snow cover, Delhey^11^ found darker coloured birds in colder regions when analysing the achromatic plumage variation of 551 species of Australian landbirds. To date, only few studies have fully explored ecotypic variations due to changing climates^12^. Local-scale studies of individual species have contributed to the overall argument that climate change is having effects, but longitudinal studies of groups of organisms or entire communities across large time scale are still largely lacking. However, they are essential to improve our understanding of how organisms are adapting to the new environmental conditions, either through phenotypic plasticity or evolutionary changes.

**Figure 1:**
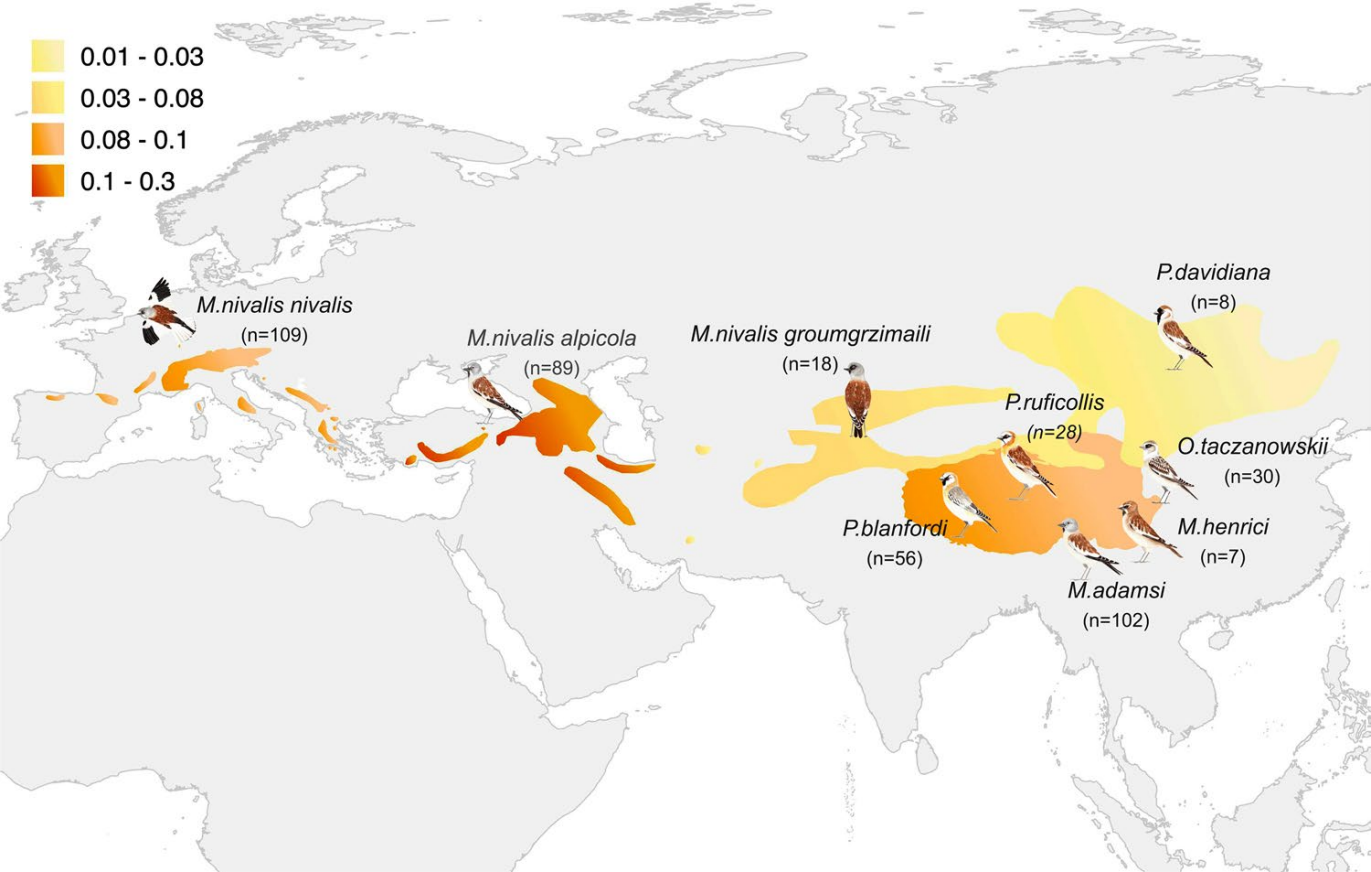
Spatio-temporal patterns in climatic data along the distributional range of the different snowfinch species (genera *Montifringilla*, *Pyrgilauda* and *Onychrostruthus*)^47^. Orange tonalities represent the increase of mean temperature (°C/10 years, estimated as the slope of the linear regression of the mean temperature over the years) in the different areas. Temperature data retrieved from the Global Historical Climatology Network^46^ (GHCN-Monthly version 2, http://www.ncdc.noaa.gov/). Numbers in brackets represent the specimens of each species measured in the collections. Drawings: Giulia Bombieri.

To explore whether and to what extent morphological traits and melanin-based colouration change in response to current climate variability, we used a 100-year-long time series (from 1850 to 1950) of 9 species/subspecies of snowfiches (Passeridae: *Montifringilla*, *Pyrgilauda* and *Onychostruthus*), out of the 17 subspecies of this group (see Fig. 1). Snowfinches are interesting species because they occupy breeding habitats in alpine ecosystems at extremely high elevations (ranging from 1500 to 5000 m; see Fig. 1), where climate change is acting fast^13,14^. Alpine regions are particularly affected by climate change^15^ and species already occupying high-altitude areas have little scope for up-slope range shifts^16^. Climate change has made the distribution range of snowfinches increasingly warmer during the last decades (Fig. 1) and, as for many organisms inhabiting alpine environments, little is known about how they are responding to climate change^17^.

## Results and Discussion

### Morphological trait changes

**Figure 2:**
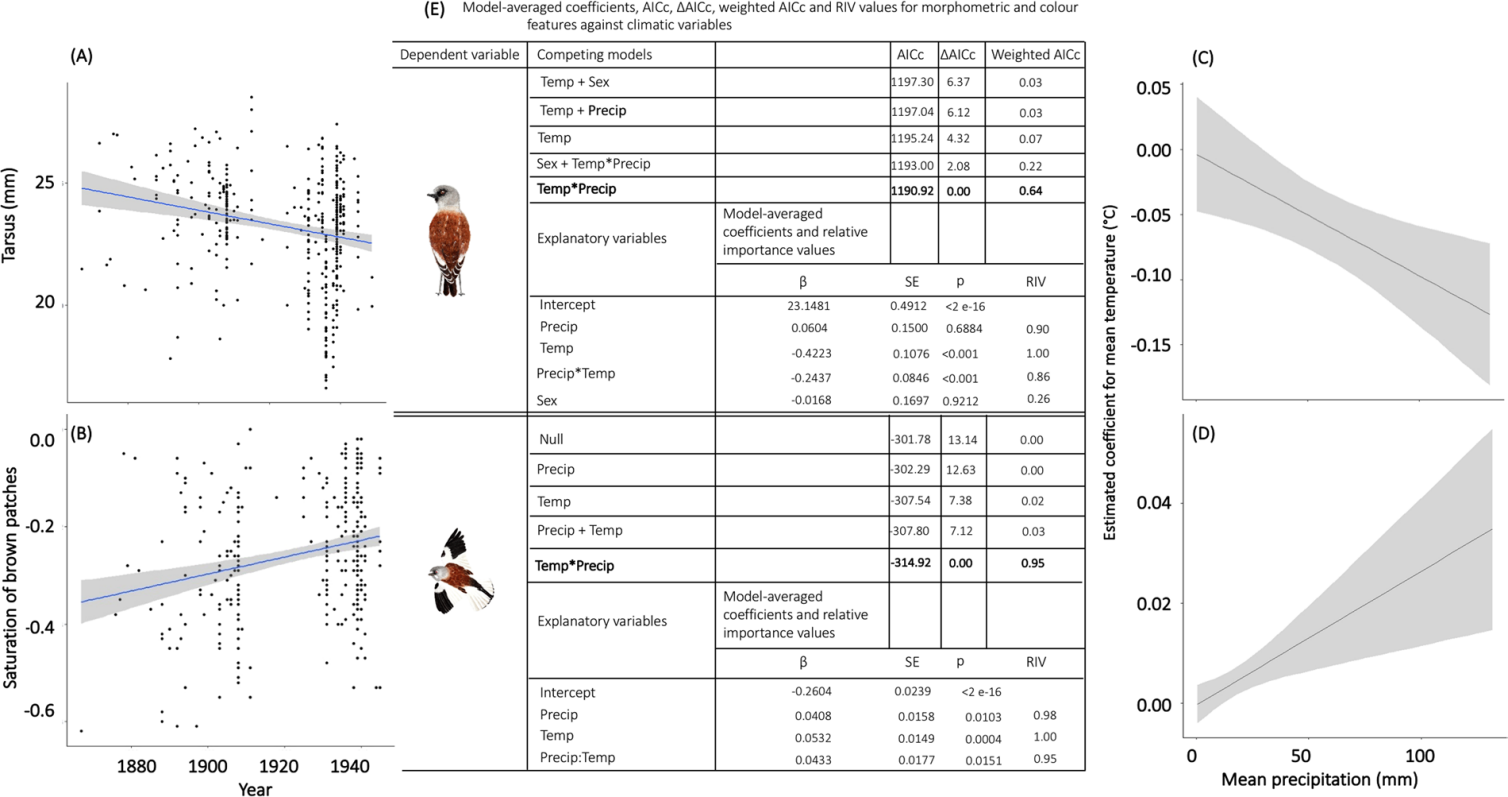
(**A**) Temporal patterns of tarsus length and (**B**) saturation of brown feathers (bottom panels); Plots of marginal effects of the interaction between mean temperature (Temp) and mean precipitation (Precip) on the variation of (**C**) tarsus length and (**D**) saturation of brown feathers. In the analyses, we used the full range of data we collected, corresponding the missing years to those were snowfinches were not found in the visited Natural History Museums and collections. (**E**) Comparison of the first five candidate models built to study the variation in tarsus length and saturation of brown feathers by mean temperature and mean precipitation. A summary of model-averaged coefficients, Akaike information criterion corrected for small sample sizes (AICc), the difference between AICc (ΔAICc), Akaike weights (weigthed AICc) and the relative importance (RIV) values is shown for those candidate models. Candidate models are ranked from the highest to the lowest (best model) AICc value. Study species in the caption: *Montifringilla nivalis nivalis*. Drawings: Giulia Bombieri.

Our first objective was to assess temporal trends in morphometric features by fitting separate general linear mixed models (GLMMs) for each morphometric trait with year as a covariate. As we had unbalanced data among species, and repeated measures within species, our models included the species as a random factor. We observed that body size of snowfinches, measured as tarsus length, has decreased through time (Fig. 2A and Table S4). We did not observe any significant temporal variation in wing length (Table S4). Tarsus length variation tracked climatic conditions over the study period (conditional R2 = 53%), being negatively related to the mean temperature but positively related to the mean precipitation (Fig. 2E). The relative importance value (RIV) of both temperature (1.00) and precipitation (0.96) in explaining morphological trends are very high (Fig. 2E). Therefore, reductions in snowfinch size are likely to be more pronounced in areas where temperature has increased and precipitation has decreased (Fig. 2C). Previous studies using principal component analysis of skeletal measurements have found that tarsus length is well-correlated with skeletal size, whereas wing length is a less appropriate measure^18^. In addition, changes in wing length may be partially due to an apparent conflict between two geographically varying selection pressures, i.e. while changes in wing length should decrease in association with increasing temperatures according to Bergmann’s rule, the same measurement is expected to increase under the same conditions according to Allen’s rule^19^. Therefore, the interpretation of changes in wing length to changing climatic conditions is potentially more complicated than interpreting variation of tarsus length.

A number of studies have provided evidence for a reduced individual size as a response to global warming over the past century^20^. Different mechanisms behind body size reductions have been proposed^7^. Sheridan and Bickford^20^ proposed that body size changes will be mediated by to two ecological factors, namely water and nutrient limitation that, ultimately, may lead to evolutionary responses favouring smaller individuals. However, whether body size shifts reflect local adaptation or phenotypic plasticity is still an open debate. Direct evidences for evolutionary responses to climate change are scarce, especially because for most taxa there is no information on which are the relevant genes encoding body size^7^. Thus, further studies isolating the ultimate and proximate causes, and potentially providing insights into the physiological consequences of changes in body size, are important to improve our understanding on the potential of species to respond to climate change. Body size is a key biological parameter^21^, influencing many aspects of an individuals’ life from the rates of energy acquisition and expenditure to the surface-to-volume ratio and food size. A change in size is thus likely to have important physiological and ecological consequences for an animal^22^. Yet, the fact that tarsus length and climate change are correlated provides no evidence on the underlying mechanisms. For example, it might be possible that variation in size is directly related to climate change, but it can also be that body size variation is related to changes in resource availability that depends on climate conditions across different geographical regions^23^. We observed that bill length, a morphometric feature highly related with changes in diet^24,25^ (Fig. S2), has also decreased through time. However, when relating variation in bill length with climatic factors, the models had generally low support (Fig. S2E), suggesting that the cause of variation in snowfinch size may not be entirely explained by changes in food resource^10,24^. An alternative hypothesis is that body size variations may be related to changes in the metabolism rates^26^. The metabolic rate exponentially scales with body mass and temperature^27^. For cold adapted alpine bird species, an increase in their metabolic rates due to an increase of ambient temperature may cause a severe decrease in their performance. By decreasing the body size, snowfinches might be compensating the expected increase of their metabolisms associated with the current warming trend observed in the alpine areas (Fig. 1).

### Melanin-based colouration changes

Our next objective was to understand whether and to what extent melanin-based colouration changes might have occurred in response to climate change. Melanin-based colouration, the most common pigment in both vertebrates and invertebrates, is associated with many important biological functions and influences individual fitness via their influence on reproduction and survival through process that include thermoregulation, energetics, fecundity, sexual selection, competition and predation, social dominance, habitat choice and pathogens^3,6,28,29^. Genes implicated in the production of melanin pigments have numerous pleiotropic effects on the resistance to various stressful factors by regulating many physiological and behavioural functions^6^. By following the same statistical approach as above, we found that the saturation levels of black and brown colours in the plumage patches of snowfinches have significantly increased through time (Fig. 2C and Table S4). We, however, did not observe any significant change of the brightness level of black and brown colours in the plumage patches of snowfinches (Table S4). The increase in saturation levels of snowfinch melanic features was positively related with mean temperature and mean precipitation (Fig. 2E; conditional R2 for saturation of brown and black patches = 31% and 9%, respectively). The relative importance value (RIV) of temperature was very high for the saturation of black (0.99) and brown (1.00) patches, whereas the RIV of precipitation was high for the saturation of brown patches (0.98) but moderated for the saturation of the black patches (0.60). Consequently, the effect of temperature on the levels of brown and black saturation increased when increasing levels of precipitation (Fig. 2D), but this pattern was only significant for the saturation of brown patches (Table S4).

Our results are in line with some previous studies suggesting that darker individuals should be favoured under warming conditions^16,29^. For instance, darker coloration could enable high-elevation populations to capitalize warming due to better radiation protection and thermoregulation, better ability to detoxify pollutants and/or higher competitive abilities^3^. In addition, as recently suggested by Delhey^11^, darker plumage could confer feather protection to degradation by bacteria, which seems to be more active in humid conditions. Therefore, in environments where individuals are able to deal with the energetic costs associated to melanin pigmentation, darker individuals might have a selective advantage^3,12^. However, as melanin-based pigmentation determine the individual ability to absorb solar radiation and gain heat, some studies have confirmed that the physiological performance of dark-pigmented endotherm animals may be constrained under high temperatures^29,30^, and therefore increase the probability of extinction^31^. These differences highlight the need for improving our understanding on how the high diversity of phenotypes produced by melanin-based pigmentation may determine individual physiological responses as an important mechanism for animals facing global warming conditions. Importantly, recent studies using time series of museum specimens have demonstrated that other environmental factors (e.g. atmospheric black carbon^32^) and anthropogenic interventions may induce phenotypic change in animal populations^33^. For instance, Nearctic horned lark *Eremophila alpestris* specimens collected in the Imperial Valley from 1984 to 2014 have darker backs, napes, and crowns than birds collected from 1918 to 1934, a change that the authors related to historical land-use changes^34^. This might well apply to all species inhabiting the Tibetan Plateau, where there has been substantial land-cover change over the last 50 years, including permafrost and grassland degradation, urbanization, deforestation and desertification^35^. Finally, the fact that we only observed an effect of climate conditions on the saturation but not on the brightness levels of melanic patches suggests that the resulting patterns of melanin-based colouration due to changes in climatic conditions might be complex and variable, as its intensity does not seem to be as much affected as its purity. More research is needed to clarify the link between melanin and individual quality^36^, and whether the variable effects that climatic conditions might have on the reflectance properties of the melanin-based colouration (e.g. brightness or saturation) are likely to affect that relationship.

Understanding how living organisms adapt to changing environments is of fundamental importance for predicting population and community resilience to climate change^37–39^. Dispersing away from deteriorating habitat is one of the most recognisable responses to environmental change^40^. Yet, escape is not always possible for all organisms, such that genetic evolution to the new local conditions and phenotypic plasticity are key adaptive responses for maintaining many aspects of biodiversity in a changing world^41^. This might be especially the case in alpine environments. To date, there have been very few cases answering the question of whether ecotypic changes represent genetic responses (i.e. evolution) or phenotypic plasticity in response to changes in weather conditions^42^. Documenting genetic change requires testing for changes of allele frequencies in genes functionally linked to ecotypic traits, or to apply quantitative genetic approaches to estimate trait heritability^7,9,42^. Yet, the relevant genes that encode ecotypic traits in snowfinch (sub)species are still unknown. As in most other species, we expect multiple genes to be involved, which adds another piece of difficulty to the task. More studies are needed for improving our knowledge on the genetic basis of colour variation in order to understand the evolution of colour phenotypes^42^, especially under global warming conditions.

## Conclusions

The correlation of changes in temperature and precipitation with shifts in body size and melanin-based colouration supports the ecogeographic rules^1,2^, with smaller individuals found where climates are becoming warmer and drier, and darker ones where temperature and precipitation are arising. Previous studies have suggested that ecotypic changes are detectable on a 100-year scale^8,33^, matching the time scale considered in this study, and have highlighted the essential role that museums and collections have for studying ecotypic responses to climate change^8^. Studies like the present one would not be possible without the efforts of myriad of persons working for the collection and preservation of this invaluable material.

Even though the present study does not identify mechanisms, it points to a clear hypothesis amenable about the adaptive role of ecotypic responses to changing environments to be tested by e.g. extensive translocation experiments: for example, that over time local organisms should be better attuned to the local climatic conditions (and express different allele frequencies) than individuals from elsewhere, as reflected in higher fitness. Doing so is, however, logistically challenging, especially in alpine environments. A more fruitful approach might be, therefore, to test the temporal changes in genetic intraspecific variability of temperature-related ecotypic traits that are linked to fitness, as has been elegantly shown in the tawny owl (*Strix aluco*)^9^. Understanding whether intraspecific variability in ecotypic traits displays higher genetic diversity would allow to further explore the consequences for population persistence of the potential ecotypic changes (i.e. to darker and smaller birds, in our case) due to selection under global changes^42,43^. As ecotypic responses to climate change could be far-reaching for animal population and community resilience to climate change, future assessments evaluating the mechanistic causes of animal ecotypic changes are urgently needed to set up positive strategies to diminish the present and future negative effects of climate change.

## Methods

We visited natural history museums and collections worldwide (Table S1) to quantify temporal changes in size and melanin-based colouration in 447 skin specimens of snowfinches. For each specimen, we measured the spectral reflectance of two melanic patches (Fig. S1), corresponding to the black and brown coloured upper parts. Previous studies have demonstrated the biological significance of melanin-based colouration, as it might affect fitness via their influence on reproduction and survival through process that include thermoregulation, energetics, fecundity, sexual selection, competition and predation^3,6,28,29^. To analyse the variation of melanin-based colouration, we needed to extract variables that summarise the chromatic variation captured in the spectral reflectance. One of the most commonly used method consists on computing indices that describe the spectral shape^44^. We computed the saturation and the brightness of the melanin-based colouration, which represent different aspects of the chromatic variation. In particular, saturation refers to the spectral purity of a colour, and brightness refers to the perceived intensity of a stimulus. To explore the possibility that the age of museum specimens contributed to colour differences^45^, we selected three species of snowfinches inhabiting areas where temperature has increased at a different rate (Table S2), and regressed colour features against the year. We selected the same period of time, from 1902 to 1945, for which we had data for all these three species. Even though the selected individuals were collected and stored during the similar time period, their colour features have shifted at dissimilar rates (Table S2). The observed shift of colour features was indeed related to the shift in temperature. Thus, we excluded the possibility that differences in plumage colouration might have resulted from degradation over time. In addition, as a surrogate of body size, we measured tarsus, wing and bill lengths of each bird, and from the specimen’s label we took the sex (when available, see Table S1) as well as the locality where the specimen was collected. Researchers are generally not in agreement on what single morphometric measurement would best reflect body size, if any^19^. Previous studies have shown that body size is best quantified using a combination of skeletal measurements, being wing and tarsus length often considered^10,18^. However, morphometric variables might show an allometric relation, changing the proportion of these traits with size. We assessed the extent of allometry between tarsus and wing length within species and found a non-significant coefficient, with slope being smaller than 1 (0.019 ± 0.012[SE]). Hence, the allometry effect was either absent or small. We first performed phylogenetic analyses to account for the relatedness of species. Even though snowfinches differ in their morphological and melanic colour features (Fig. S1), the structure of the phylogeny alone did not explain the observed variation in morphometric and colouration traits (Table S3). To evaluate the effect of climate change, and using the spatial coordinates of each specimen, we selected the closest weather station (data retrieved from the Global Historical Climatology Network (GHCN-Monthly version 2, http://www.ncdc.noaa.gov/)^46^ to determine mean temperature and precipitation data per year during the same time window for which we had species-specific data.

Additional methods, including data collection and its accessibility, statistical analyses, additional results and references, are available in the Supplementary Information.

## Supplementary information

Supplementary Information

## Data Availability

The datasets supporting the conclusions of this article are included in the Table S1.
