# Supplementary material for: Ecotypic changes of alpine birds to climate change
Source: Sci Rep. 2019 Nov 6;9:16082. doi: 10.1038/s41598-019-52483-0 (PMC6834662; doi:10.1038/s41598-019-52483-0)

Supplementary Information

Ecotypic changes of alpine birds to climate change

Maria del Mar Delgado[1a], Chiara Bettega[a], Jochen Martens[b] and Martin Päckert[c]

[a]*Research Unit of Biodiversity (UMIB, UO-CSIC-PA), Oviedo University - Campus Mieres, 33600 Mieres, Spain*

[b]*Institut für Organismische und Molekulare Evolutionsbiologie, Johannes Gutenberg-Universität, 55099 Mainz, Germany.*

[c]*Senckenberg Natural History Collections, Museum für Tierkunde, Koenigsbruecker Landstraße 159, 01109 Dresden, Germany*

Material and methods

Data collection

We measured 447 skin specimens of nine species of snowfinches (5 species/subspecies of genus *Montifringilla*: *M. nivalis nivalis* (n = 109), *M. nivalis alpicola* (n = 89), *M. adamsi* (n = 102), *M. henrici* (n = 7) , *M. groumgrzimaili* (n = 18); 3 species of genus *Pyrgilauda*: *P. ruficollis* (n = 28), *P. blanfordi* (n = 56), *P. davidiana* (n = 8); and 1 species of genus *Onychostruthus*: *O. taczanowskii* (n = 30)) from ten countries of their distribution ranges (see Fig. 1; Table S1) that have been preserved in the following natural history museums and collections: Museo Civico di Zoologia (Rome, Italy), MUSE (Trento, Italy), Naturhistorisches Museum Wien (Vienna, Austria), Museum für Naturkunde (Berlin, Germany), Zoologische Staatssammlung München (Munich, Germany), American Museum of Natural History (New York, USA) and The Field Museum of Natural History (Chicago, USA).

Collection dates ranged between 1806 and 2004, but most of the specimens were dated from 1850 to 1950. They were not exposed specimens, and they were always preserved in opaque closets and in dark environment, thus preventing the plumage degradation due to light. Any specimen showing a poor conservation state (i.e., poor feather integrity or visible presence of chemical products on the surface; see[40]) was

directly not measured. We measured the spectral reflectance of specimens using an Ocean Optics Jaz spectrometer and PX-2 flash lamp (Ocean Optics, Dunedin, FL); see[43] for a review of colour measurement techniques. Our reflectance probe was mounted in a black rubber probe holder, which excluded all external light and maintained the probe at a fixed distance (5 mm) from, and oblique to, the feather surface. For each bird, we measured the reflectance of two melanic patches (Fig. S1A and Fig. S1C), corresponding to the black and brown coloured feathers on the upper surface of the wings and back, respectively. We recorded three measurements per patch, each of which comprised an average of 10 readings collected in rapid succession by the operating software[40]. Because the patches were of similar colouration within specimens, we averaged the three measurement per patch together. All reflectance data are expressed as the percentage of reflectance from a white standard (WS-1, Ocean Optics). In addition, we measured the forearm, wing, tail and bill length of each bird using a digital calliper (± 0.1 mm), and collected from the specimen's label the sex and age (when available), as well as the locality where the snowfinch came from.

Colourimetric variables

We calculated a series of colourimetric variables in order to quantify the colour of the 300–700 nm interval (which accounts for the entire visual sensitivity range of birds) for all recorded spectra. Whenever negative reflectance values were present, these were corrected by adding the absolute value of the most negative value to the whole spectra, hence preserving the shape of the curve while forcing the minimum value to zero. Negative values in spectra are uninterpretable, but unfortunately quite common besides doing the best to properly calibrate the spectrophotometer. To deal with this problem, Maia et al.[44] included two different ways: (1) adding the absolute value of the most negative value to the whole spectrum, in which case the shape of the curve is preserved, but the maximum reflectance is much higher or (2) changing all negative values to zero with zero, which preserves absolute reflectance values, but may cause the spectral shape to be lost. The best transformation varies according to the final goal of the analysis. Because (a) the source of the negative values in our case most probably came from the spectrophotometer; and (b) we did not use the brightness intensity but its mean (in which case it is more important to preserve the total shape of the curve),

we added the absolute value of the most negative value to the whole spectrum as the best solution suggested by the authors. This option allowed us to preserve the total shape of the curve, which is more important for our selected colourimetric variables. We then averaged the three readings collected within each colour patch of each individual's plumage. Electrical noise from the spectrometer was removed by applying local polynomial regression fitting or loess smoothing to the averaged spectra. For this purpose, we used a span of 0.05 as this allowed us to remove the noise while maintaining the spectra original shape.

For each of the two coloured patches, we estimated objective spectral colorimetric variables[43], including two standard descriptors of reflectance spectra: the mean brightness) and saturation. We determined mean brightness as the mean reflectance over the visual range of the smoothed spectra. Spectral saturation was determined as the relative reflectance between the area around the peak with reflectance greater or equal to half of the peak reflectance. All colorimetric variables were calculated using the package pavo[44] for R version 3.2.2 for Windows[45].

Climate data

In order to evaluate the effect of climate change, and using the latitudinal/longitudinal coordinates of each specimen, we selected the closest weather station (data retrieved from the Global Historical Climatology Network[41] (GHCN-Monthly version 2, http://www.ncdc.noaa.gov/)) to determine mean temperature and precipitation data per year during the same time window for which we had species-specific data. The GHCN database provides historical temperature and precipitation data for thousands of land stations around the world. The period of record varies from station to station, but the average temperature record is going back to 60 years long, while more than 1600 records are greater than 100 years.

Data analyses

For phylogenetic analysis we reconstructed a phylogeny of snowfinches based on four loci (mitochondrial cytochrome-*b* [cyt*b*: 862 bp] and NADH dehydrogenase subunit 2 [ND2: 1041 bp]; nuclear introns myoglobin [myo: 724 bp] and ornithine-decarboxylase [ODC: 611 bp]). We complemented a preliminary snowfinch data set from a previous

study[46] with further newly generated sequence data for a total sampling of seven currently recognized snowfinch species. Since intraspecific genetic variation has been described for the white-winged snowfinch (*Montifringilla nivalis*)[47] we included sequence data for three different subspecies (*nivalis*, *alpicola*, *groumgrzimailii*) in our data set. For hierarchical outgroup rooting we added one sample of the closest relative of snowfinches, the rock sparrow (*Petronia petronia*) to our data set and sequence data of a more distantly related species, the tree sparrow (*Passer montanus*; for origin of samples and accessibility of sequence data see Table S5). DNA extraction, PCR settings and sequencing followed the protocols in[46]. The sequences were aligned with MEGA 6.06[48] and the alignments (a total of 3238 bp) were cross-checked via inspection of the sequence chromatograms. All newly generated sequences were deposited at GenBank under accession numbers MM337349-MM337381.

A multi-locus tree was reconstructed with BEAST v.1.8.1[49]. When mitochondrial markers were additionally partitioned by codon, pilot runs with different partition schemes yielded poor Effective Sample Size (ESS) values for posterior and tree prior (checked with Tracer 1.4[50]). This was apparently due to extremely low means (and consequently poor ESS values) for several substitution rates which typically occur in small data sets (simply because the respective substitutions at a given codon position do not occur in the data set). We therefore applied a simple partitioning by gene (four partitions) to our data set (avoiding overpartitioning[51]) and used MrModeltest for estimation of the best fit model for each of the four partitions (for model settings see Table S6). We ran BEAST for 50 million generations (trees sampled every 5000 generations) under the uncorrelated lognormal clock model for all loci with the 'auto-optimize' option activated and a birth-death process prior (with incomplete sampling assumed) applied to the tree. Trees were summarized with TreeAnnotator v1.4.8 (posterior probability limit = 0.5) using a burn-in value of 3000 (trees) and the median height annotated to each node. For this run ESS values were higher than 200 for all parameters.

For time calibration of the Bayesian tree we applied an empirical substitution rate of 0.0105 substitutions per site per lineage per Million years to the cyt*b* partition [52]. We furthermore applied a secondary calibration to the node uniting snowfinches (*Montifringilla*, *Pyrgilauda* and *Onychostruthus*) and rock sparrows (*Petronia*) assigning

a fixed node age of 11.4 Myr as an estimate of the time of the most recent common ancestor (tmrca prior set to a normal distribution, SD= 1.0) inferred from a fossil calibration of the Passerida phylogeny by Päckert et al.[46]. To validate the phylogeny obtained from BEAST we also calculated a maximum likelihood (ML) tree with RAxML v7.2.6 [53] (using the GUI PYTHON application v0.93[54]). ML bootstrap support was obtained using the thorough bootstrap option with 1000 replicates under the GTR+Γ+I model.

The BEAST tree and the RAxML tree were fully concordant (Fig. S1B). All nodes received strong support except of the sister-group relationship of *Onychostruthus* and *Pyrgilauda* and of the node uniting the three subspecies of *Montifringilla nivalis*. In contrast to single-marker analyses[55], monophyly of *Pyrgilauda* was fully supported in our phylogeny. Our divergence time estimates were twice as old as those inferred by Lei et al.[55] (Fig. S1B) from their snowfinch phylogeny. This divergence is due to the fact that Lei et al.[55] had applied "a divergence rate of 2% per million years for the cytochrome b gene" and must have converted this estimate in a rate value of 0.02. If their calibration was based on that rate estimate, it would be problematic, because 2% per Million years is an empirical value for divergence between clades, whereas time calibration with BEAST relies on a substitution rate per lineage, which is exactly half of the divergence between clades (0.0105 substitutions per site per lineage per Million years, see[52]).

We used the time-calibrated BEAST tree for further analysis. We first performed phylogenetic analyses which included (1) morphometric parameters and (2) melanic-based colours as response variables to account for the relatedness of species. We estimated the phylogenetic signal of colour features using the phylosig function in PHYTOOLS package in R[56] that specifically allows to compute Pagel's λ incorporating sampling error[57], whose value ranges continuously from 0 to 1. λ values close to 1 are indicative that the structure of the phylogeny alone can explain colour variation. Instead, a λ value next to 0 indicates that colour variation is random regarding the phylogeny.

We then studied the temporal shift of each morphometric and melanic feature by fitting general linear mixed models (GLMMs) with the feature recorded (i.e. morphometric: tarsus, wing and bill lengths; melanin-based colouration: brightness and

saturation) as response variables and year as a covariate. As all variables were normally distributed, we used normal distribution. As we had repeated measures within species, we included species as a random factor. Further, we built additional GLMMs to explore whether the temporal variation in morphometric and melanin-based colour features (when significant) was related to climatic variables. As candidate climatic variables we included two weather covariates, namely mean temperature and precipitation. We assessed collinearity using the Variance Inflation Factor (VIF) among mean temperature and mean precipitation but, as the values of these predictors were close to 1, none of them were omitted from the models[58]. Exploratory analyses revealed sex differences in morphometry, but not in melanin-based features, within species. We thus included sex as a factor in the models for morphometric features in order to account for differences related to sexual dimorphism. All variables, other than categories, were standardized using a z-score transformation with a mean of 0 and a standard deviation of 1.

Once we generated the sets of competing models, we employed a model averaging on the 95% confidence set to derive values of AICc, ΔAICc, Weighted AICc, parameter coefficients and the relative importance values (RIV) of each explanatory variable using the full-model averaging approach to select the best competing model. We considered models with ΔAICc values lower than 2 as equally competitive. When there might be high model selection uncertainty, model averaging allows formal inference based on the entire set of models[59,60], in our case, the 95% confidence set. Parameter estimates produced by model averaging derive from weighted averages of these values across all models in the set considered[60]. In particular, the relative importance value (RIV) of each explanatory variable is calculated by summing Akaike weights across all models which contain the variable[61]. All analyses were performed using R version 3.2.5 statistical software R Core Team[45]. GLMMs were run using the “lme4”[62] and “nlme”[63] packages. Multimodel inference and model averaging were run using the “MuMIn”[64] package.

Supplementary Tables

Table S1. Excel file with data ordered by (A) species/subspecies. Columns corresponds to (B) bill length (mm); (C) tarsus length (mm); (D) wing length (mm); (E) sex: *F* for

females and *M* for males; (F) age: *ad* for adults and *juv* for juveniles; (G) country; (H) year; (I) museums; (J) brightness of black patches; (K) saturation of black patches; (L) brightness of brown patches; and (M) saturation of brown patches.

Table S2. We explored whether age of museum specimens might be contributing to colour differences by selecting three species of snowfinches inhabiting areas where temperature has increased at a different rate, and regressed colour features against the year. We selected the time period, from 1902 to 1945, for which we had data for all these three species. The different columns represent the Estimate (*β*) of the regression models. S denotes the saturation and B the brightness of the melanic colours.

β Temperature	*β* S brown	*β* S black	*β* B brown	*β* B black	Species
0.18	-0.0007	-0.0001	0.02627	0.0441	*M. nivalis nivalis*
0.47	0.0021	0.0031	0.0508	-0.0167	*M. nivalis alpicola*
0.21	-0.0021	0.0011	0.1542	-0.0002	*M. adamsi*

Table S3. Summary statistics from the phylogenetic analyses of morphometric and melanin-based colour features.

			λ	p
Morphometric features		Tarsus length	0.0000	0.99
		Bill length	1.0254	0.31
		Wing length	1.0254	0.07
Melanin-based colour features			λ	p
	Brightness	black	0.8770	1
		brown	0.4926	1
	Saturation	black	0.2131	1
		brown	0.3667	1

Table S4. Summary of models (GLMMs) assessing temporal trends in morphometric traits and melanic-colour features. Only when the temporal change in morphometric and melanin-based features was significant (features highlighted in bold), it was possible to explored whether that change was related to climatic variables.

			β	*SE*	*p*
**Bill length**		Intercept	12.27	0.54	<0.001
		sex(male)	-0.13	0.12	0.20
		year	-0.27	0.06	**<0.001**
Wing length		Intercept	104.97	3.45	<0.001
		sex(male)	2.82	0.62	<0.001
		year	-0.15	0.34	0.66
**Tarsus length**		Intercept	23.11	0.57	<0.001
		sex(male)	0.03	0.16	0.86
		year	-0.23	0.09	0.01
Black	Brightness	Intercept	7.40	0.29	<0.001
		year	-0.53	0. 29	0.07
	**Saturation**	Intercept	-0.27	0.01	<0.001
		year	0.02	0.01	**0.006**
Brown	Brightness	Intercept	13.68	1.39	<0.001
		year	0.72	0.38	0.06
	**Saturation**	Intercept	-0.26	0.02	<0.001
		year	0.03	0.01	**<0.001**

Table S5. Origin of samples and accession numbers of sequences used for phylogenetic analyses (outgroup *Passer montanus* from Zuccon et al.[65]); collection acronyms: MAR= own samples J. Martens at SNSD; NRM= Natural History Museum of Stockholm, Sweden; UWBM= Burke Museum of Natural History and Culture, Seattle, USA.

sample no	species	country	cytb	ND2	myo2	ODC
MAR2212	*Montifringilla adamsi*	China	MN337349	MN337357	MN337368	MN337374
MAR1775	*Pyrgilauda blanfordi*	China	MN337350	MN337358	MN337366	-
MAR2093	*Pyrgilauda davidiana*	China	MN337351	MN337359	MN337367	MN337375
MTD2009-160	*Montifringilla nivalis nivalis*	Italy	KX109628	KX109703	KX109668	KX109742
MAR1532	*Montifringilla nivalis alpicola*	Russia	MN337352	MN337361	MN337370	MN337377
MAR3111	*Montifringilla nivalis groumgrzimaili*	Mongolia	MN337353	MN337362	MN337371	MN337378
MAR2204	*Montifringilla henrici*	China	DQ244059	MN337360	MN337369	MN337376
MAR2206	*Pyrgilauda ruficollis*	China	MN337354	MN337363	AY228306	GU816915
MAR426	*Onychostruthus tazcanowskii*	China	MN337355	MN337364	MN337372	MN337380
NRM 976359[65]	*Passer montanus*	Sweden	AY228073	GU816845	AY228311	DQ785937
UWBM66486	*Petronia petronia bervirostris*	Mongolia	MN337356	MN337365	MN337373	MN337381

Table S6. Substitution models estimated with MrModeltest for the two mitochondrial markers (Cytb, ND2) and the two nuclear introns (myo, ODC); base frequencies: πA, πC, πT, πG; α: gamma shape parameter; I: proportion of invariable sites; kappa: transition/transversion ratio; substitution rates (with R [G-T]= 1.0): R(a) – R(e).

	Cytb	ND2	myo	ODC
bp	862	1041	673	680
	HKY+I	GTR+G	K80+I	GTR
πA	0.2869	0.3115	1.0000	0.2793
πC	0.3745	0.3592	1.0000	0.1782
πT	0.1292	0.1089	1.0000	0.1982
πG	0.2094	0.2205	1.0000	0.3443
α	-	0.1441	-	-
I	0.5074	-	0.6334	-
kappa/Ti/Tv ratio	6.985	-	1.8341	-
R(a)[A-C]	-	0.4972	-	10.2996
R(b)[A-G]	-	29.7615	-	15.6230
R(c)[A-T]	-	0.2509	-	2.9454
R(d)[C-G]	-	0.3121	-	6.9234
R(e)[C-T]	-	11.1814	-	15.6436

Supplementary Figures

Figure S1. (A) Melanic patches of snowfinches, which have pale brown upperparts and black areas of their wings; (B) Time-calibrated phylogeny of snowfinches: BEAST tree, MCMC chain length of 50 Million generations, node support from Bayesian posterior probabilities above nodes and from maximum likelihood bootstrap (RAxML) below nodes; grey bars indicate 95% HPD intervals; (C) reflectance spectra of snowfinches melanic (i.e. brown and black) feathers. Drawings: Giulia Bombieri.

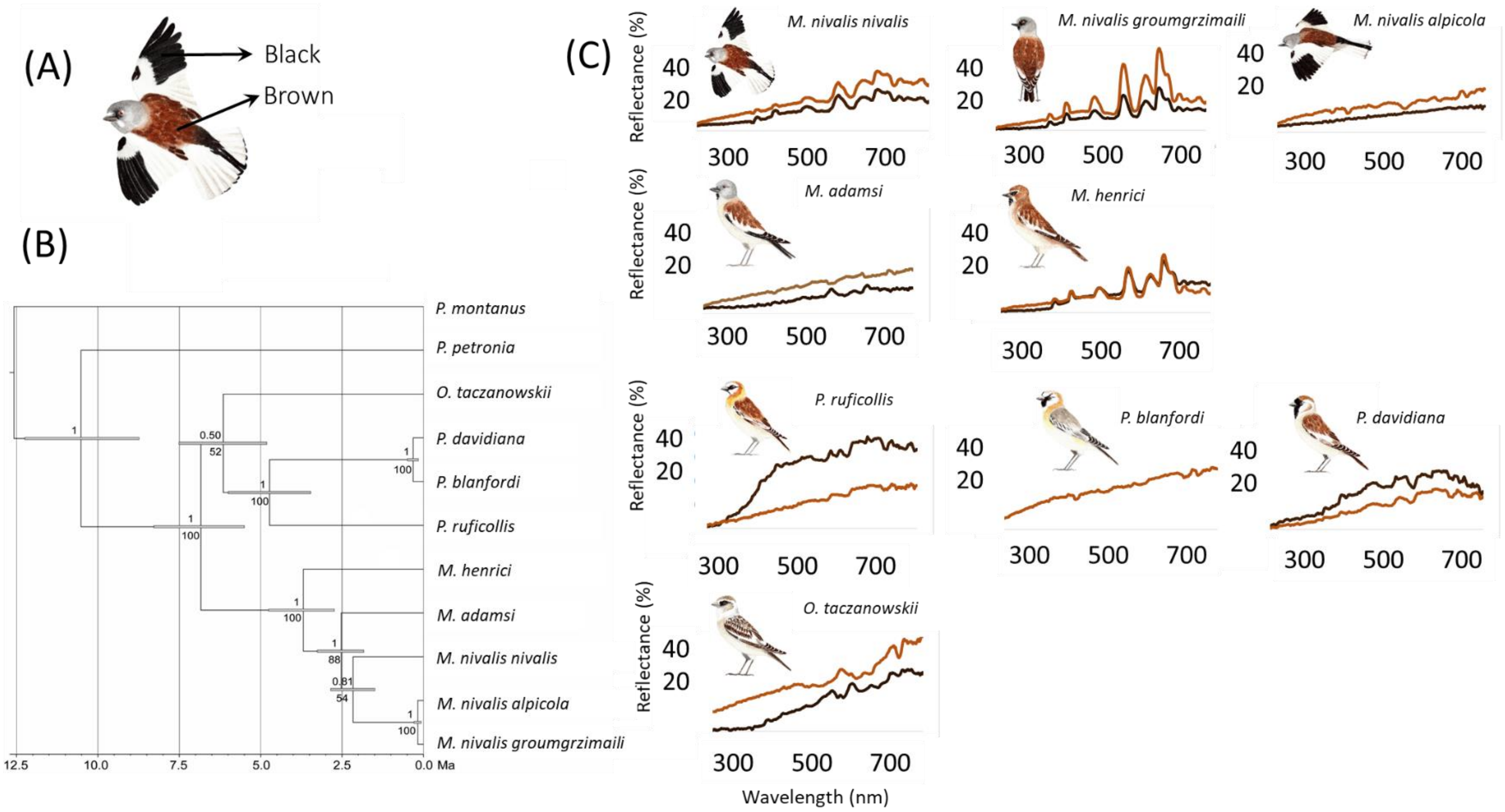

Figure S2. (A) Temporal patterns of bill length and (B) saturation of black feathers; Plots of marginal effects of the interaction between mean temperature (Temp) and mean precipitation (Precip) on the variation of (C) bill length and (D) saturation of black feathers. (E) Comparison of the first five candidate models built to study the variation in bill and length, as well as in saturation of black feathers by mean temperature and mean precipitation. A summary of model-averaged coefficients, AICc, weighted and RIV values is shown for those candidate models. Candidate models are ranked from the highest to the lowest (best model) AICc value. Drawings: Giulia Bombieri.

(A)

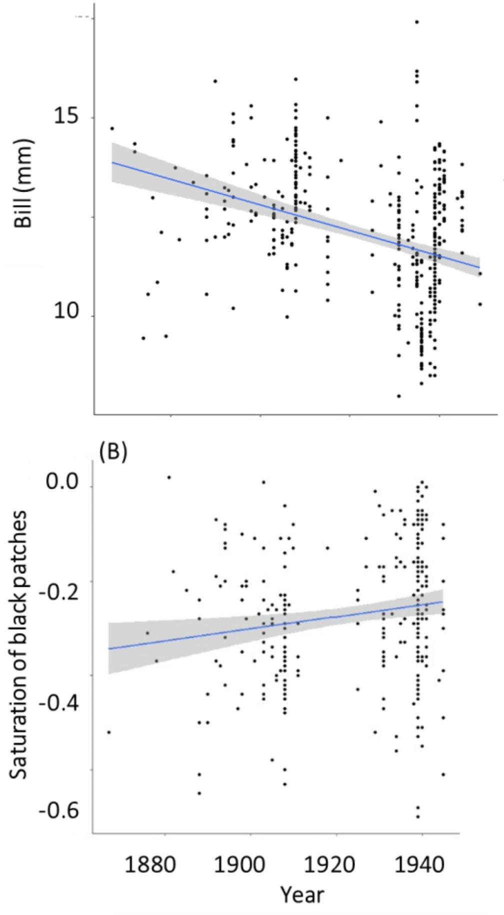

(E) Model-averaged coefficients, AICc, ΔAICc, weighted AICc and RIV values for morphometric and colour features against climatic variables

Dependent variable	Competing models		AICc	ΔAICc	Weighted AICc
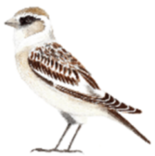	Temp		885.95	12.71	0.00
	Temp + **Precip**		874.64	1.40	0.17
	Temp + Precip + Sex		874.32	1.09	0.20
	Temp*Precip		873.48	0.24	0.30
	**Sex + Temp*Precip**		**873.23**	**0.00**	**0.34**
	Explanatory variables	Model-averaged coefficients and relative importance values			
		β	SE	p	RIV
	Intercept	11.9415	0.4785	<2 e-16	
	Precip	-0.3426	0.0921	0.0002	1.00
	Temp	-0.2749	0.0658	<0.001	1.00
	Precip*Temp	0.0919	0.0512	0.0736	0.64
	Sex	0.1570	0.1022	0.1257	0.53
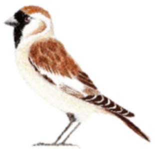	Precip		-213.78	9.09	0.00
	Null		-214.82	8.04	0.01
	Precip + Temp + Precip:Temp		-221.69	1.18	0.22
	Precip + Temp		-222.82	0.05	0.38
	**Temp**		**-222.87**	**0.00**	**0.39**
	Explanatory variables	Model-averaged coefficients and relative importance values			
		β	SE	p	RIV
	Intercept	-0.2682	0.0097	<2 e-16	
	Precip	0.0098	0.0117	0.403	0.60
	Temp	0.0427	0.0128	<0.001	0.99
	Precip:Temp	0.0038	0.0110	0.7287	0.22

(C)

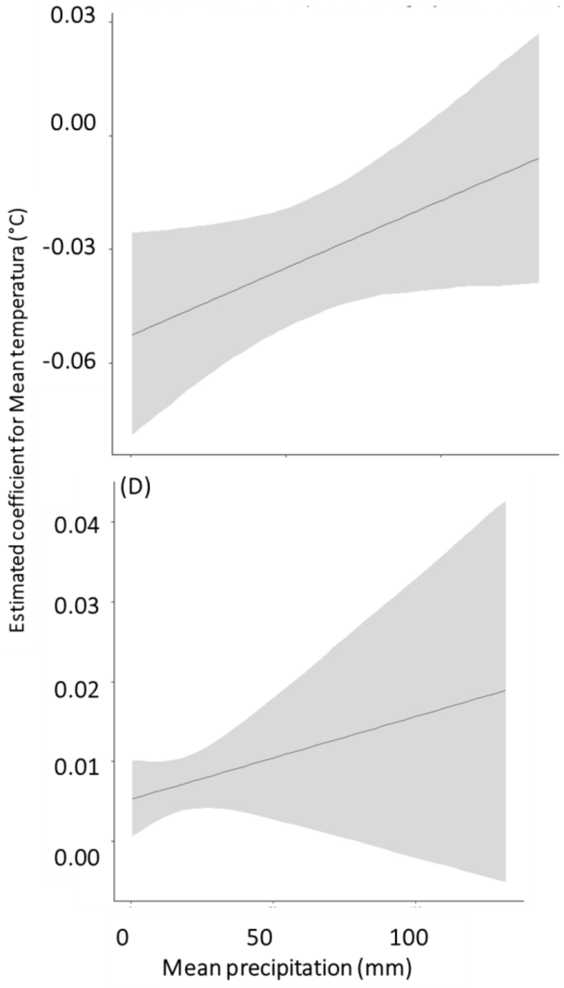

Supplement: Supplementary file 1 — Supplementary Information [file 41598_2019_52483_MOESM1_ESM.pdf]
